# Geochemical studies of natural graphites by INAA determined trace element concentrations

**DOI:** 10.1007/s10967-015-4247-6

**Published:** 2015-06-20

**Authors:** Jerzy Janczyszyn, Barbara Kwiecińska

**Affiliations:** Faculty of Energy and Fuels, AGH – University of Science and Technology, Al. Mickiewicza 30, 30-059 Kraków, Poland; Faculty of Geology, Geophysics and Environmental Protection, AGH – University of Science and Technology, Al. Mickiewicza 30, 30-059 Kraków, Poland

**Keywords:** Graphite, Trace elements, Graphitization index, Activation analysis

## Abstract

An attempt to characterize natural graphites by their trace element content is reported. Ten samples of graphite of known deposits over the world are classified by their graphitization indices, ranging from 0.26 to 1.00, and analyzed for trace elements content. Results of neutron activation analysis of 28 evaluated elements, i.e. Na, Rb, Cs, Th, U, Cr, Fe, Co, As, Sb, Ag, Au, Se, Te, Zn, Hg, Zr, Hf, Ta, Sc, La, Ce, Nd, Sm, Eu, Tb,Yb and Lu, are presented and briefly discussed.

## Introduction

Trace elements (TE) in graphite are of interest due to the practical applications of this material and the purely scientific point of view. For practical reasons mainly the pure graphite is analyzed for its use as a nuclear grade material in reactors, but also as a material for analytical chemistry applications [1–4]. Recently also its different form known as graphene is of much interest. In the case of pure science, among others, the geochemistry of different types of rocks hosting natural graphite uses TE as markers [5–7]. In this paper only the natural variety of graphite is concerned mainly for the geochemical meaning of its TE content.

## Experimental

Graphites and graphite-like organic substance were classified and characterized on the basis of optical and structural studies years ago [8, 9]. In the present work ten samples of natural graphite classified in three groups have been tested. These groups are: graphite, semi-graphite and meta-anthracite.

The first group consisted of samples of natural graphite with high graphitization index (determined according to the method defined by [10, 11] in the range of 0.56–1.00, occurring mainly in the vein deposits of metamorphic rocks from the preCambrian granulite and amphibolite facies from: Sri Lanka (nr. 2), Male Vrbno, Czech Republic (nr. 6), Passawa, Bawaria (nr. 8), Witostowice, Poland (nr. 16), Tsavo, Kenya (nr. 63) and Młoty, Poland (nr. 100).

In the second group are two samples of semi-graphite of the graphitization index below 0.56 from carboniferous deposits: Keiserberg, Austria (nr. 3) and Hurlford, Scotland (nr. 83). First semi-graphite (sample nr. 3) represents greenschist facies from phyllitic rocks and the second (sample nr. 83) comes from thermally metamorphosed (contact metamorphism) ones.

In the third group were placed two samples of meta-anthracite, also from carboniferous rocks, characterized by low degree of graphitization (below 0.37 and 0.27). The sample nr. 1 (from Czywczyn-Ukraine) formed in greenschist facies of regional metamorphism, and the sample nr. 9 (Sonora-Mexico) comes from sedimentary rocks thermally metamorphosed in contact with magmatic intrusion.

In this work we would like to present results of geochemical studies supplementing the applied so far test methods of the above mentioned graphite samples. The study was made with the use of the method of instrumental neutron activation analysis (INAA). From the practical experience gained in our laboratory from many years of analysing samples of different materials we can say that INAA allows for the determination of 20–30 elements in amounts from 0.05 ppm (for Au) to 50 % (for Fe). The reactor version of this method was applied with the use of nuclear reactor of the Institute of Atomic Energy in Świerk near Warsaw (now the National Centre for Nuclear Research). For more details of the analytical methodology see [12, 13].

In the samples, contents or their upper levels, of the following elements were determined: alkaline metals—Na, Rb, Cs, actinides—Th and U and metals—Cr, Fe, Co, As, Sb, Ag, Au, Se, Te, Zn, Hg, Zr, Hf, Ta, and Sc, as well as of the rare earth elements (REE)—La, Ce, Nd, Sm, Eu, Tb, Yb and Lu.

The following conditions of the analytical procedure were applied:

- Neutron flux density 2 × 10^13^ n/cm^2^s
- Irradiation time 3 h
- Sample mass 0.1–0.15 g
- First counting
  - Decay time from the end of irradiation to the beginning of counting 160 h
  - Counting time 500 s
- Second counting
  - Decay time 30 days
  - Counting time 2000 s

For the measurements a semiconductor Ge (Li) detector of gamma radiation was applied. It has 50 cm^3^ active volume and ~3 keV resolution (for the 1332 keV ^60^Co gamma line). The applied sample-to-detector distance was mainly 5 cm except that for standards of high activity when it reached even 20 cm. The counting statistics was different, depending on the concentration of given element in samples. Generally the total (statistical and calibration) relative standard deviation ranged from 0.5 % up to the 33 %. Accordingly, the determination limit was assumed as the concentration of element equivalent to three total relative standard deviations equal to 33 % or higher.

The following samples were used as standards:

- Mono-elemental, made by evaporation from a given volume of standard solutions of Nd, Sm, Eu, Tb, Yb, Lu, and
- multi-elemental standard reference materials: shale TB (Germany), lake sediment SL1 (IAEA), greizen GnA (Germany), copper ore CuPl (Poland), zinc ore ZnU (Poland), SOIL7 (IAEA).

The standard reference material SL1 was used as reference standard for QC.

The detailed description of the gamma spectra treatment and the calibration procedure are given in [12]. Here, only some very general information is presented for quick orientation. All calculations were performed with the system of programs called System of programs for activation analysis calculations (SPAAC) developed in our laboratory. It consists of five programs for: gamma spectra analysis (OPWID [14]), nuclide identification and overlapping peak correction (KORPIK), blank correction (FOLKOR), calibration (PCKAL [15] ) and calculation of concentrations and their standard deviations (PIKCOW [15] ). Peak searching is based on the well known Sterlinski’s algorithm yielding values of *L* (proportional to peak area) and *V* (variance of L). The ratio ${{\sqrt V } \mathord{\left/ {\vphantom {{\sqrt V } L}} \right. \kern-0pt} L}$ indicates a peak when it is smaller than the prescribed critical value (e.g. 0.25). For each peak the following values are calculated: peak center (energy), area, FWHM and FWTM. Some preliminary corrections of peak overlapping are also made. These data and other for more detailed analysis are transferred to KORPIK where peaks are prescribed to radionuclides on the basis of a prepared library. Then the energy recalibration and precise correction for peak interferences and blank is done. PCKAL and PIKCOW perform the calibration and determination steps of analysis using general model of least squares fitting. It is useful when more then one measured signal (peak) for an element is used for analysis. The final results contain concentrations and single standard deviations for each element present in standards. The system proved to be very time-effective while yielding quite accurate results of analysis.

## Results and discussion

The obtained results of analysis are shown in Tables 1, 2 and 3. The reason of poor detection limits for some elements is mainly the poor statistics of the integrated number of counts of characteristic photopeaks. The limits differ from sample to sample due to different concentration of most active radionuclide. For majority of determined elements the quantified accuracy of results is reported in the Table 4. The certified values of elements concentrations for SL1 are compared with results obtained in the applied analytical procedure.

**Table 1:** Results of the analysis of graphite samples

No.	Sample no. (graphitization index)	Content and uncertainty (ppm)			
		Na	Rb	Cs	Sc
*Graphites*					
1	2 (1.00)	<1200	<20	<0.6	0.47 ± 0.04
2	63 (0.736)	<400	<32	<0.21	2.80 ± 0.21
3	8 (0.715)	<400	<11	<0.4	1.68 ± 0.14
4	6 (0.626)	<1800	<70	7.6 ± 1.7	9.0 ± 0.5
5	16 (0.567)	1.04 ± 0.15 %	155 ± 26	2.9 ± 0.4	19.5 ± 4.3
6	100 (0.567)	0.29 ± 0.09 %	241 ± 28	9.7 ± 1.0	16.0 ± 1.3
*Semi-graphites*					
7	3 (0.555)	<700	20 ± 5	3.9 ± 0.5	1.93 ± 0.37
8	83 (0.543)	<500	<26	<0.8	6.6 ± 1.2
Meta-anthracites					
9	1 (0.371)	<700	31 ± 7	3.9 ± 0.5	10.7 ± 1.7
10	9 (0.269)	<500	34 ± 8	3.0 ± 0.3	3.22 ± 0.17

		Th	U	Fe (%)	Co
*Graphites*					
1	2 (1.00)	0.33 ± 0.10	<3.1	0.34 ± 0.02	<0.17
2	63 (0.736)	<0.9	<7	0.63 ± 0.04	1.77 ± 0.09
3	8 (0.715)	0.43 ± 0.08	<2.4	0.59 ± 0.03	2.06 ± 0.11
4	6 (0.626)	4.5 ± 0.5	11.3 ± 2.4	7.4 ± 0.4	52 ± 3
5	16 (0.567)	15.3 ± 1.3	17.7 ± 3.3	1.06 ± 0.06	6.1 ± 0.4
6	100 (0.567)	10.6 ± 0.9	15.1 ± 2.5	3.8 ± 0.2	9.4 ± 0.5
*Semi-graphites*					
7	3 (0.555)	0.39 ± 0.11	<2.4	0.33 ± 0.02	2.11 ± 0.12
8	83 (0.543)	0.95 ± 0.16	<2.8	2.44 ± 0.13	2.54 ± 0.22
*Meta-anthracites*					
9	1 (0.371)	4.7 ± 0.5	<4	1.24 ± 0.07	12.8 ± 0.7
10	9 (0.269)	3.6 ± 0.3	<4	0.61 ± 0.04	2.28 ± 0.12

		Zn	Hg	Se	Te
*Graphites*					
1	2 (1.00)	3280 ± 120	2.37 ± 0.48	<5	<12
2	63 (0.736)	<20	<0.9	2.91 ± 0.83	<6
3	8 (0.715)	<14	1.35 ± 0.22	<1.9	<7
4	6 (0.626)	<0.28 %	5.3 ± 0.9	<9	<19
5	16 (0.567)	<70	<2.1	<8	<21
6	100 (0.567)	<80	<3.1	<13	<13
*Semi-graphites*					
7	3 (0.555)	<13	0.67 ± 0.15	<2.8	<6
8	83 (0.543)	<230	430 ± 506.1	<5	<10
*Meta-anthracites*					
9	1 (0.371)	<30	±0.8	<5	<9
10	9 (0.269)	<13	1.21 ± 0.26	<2.4	<9

**Table 2:** Results of the analysis of graphite samples

No.	Sample no. (graphitization index)	Content and uncertainty (ppm)			
		As	Sb	Ag	Au (ppb)
*Graphites*					
1	2 (1.00)	1100 ± 60	50 ± 15	3.9 ± 0.7	<40
2	63 (0.736)	<7	0.53 ± 0.12	<1.1	<18
3	8 (0.715)	<5	0.90 ± 0.22	<0.7	62 ± 7
4	6 (0.626)	<11	63 ± 4	<2.7	50 ± 9
5	16 (0.567)	<13	< 0.7	<4	<27
6	100 (0.567)	<18	< 0.9	<4	<30
*Semi-graphites*					
7	3 (0.555)	24.3 ± 5.3	5.6 ± 0.7	<1.1	<24
8	83 (0.543)	<9	1.73 ± 0.57	<3.3	<27
*Meta-anthracites*					
9	1 (0.371)	<10	<1.2	4.4 ± 0.8	<30
10	9 (0.269)	<7	1.39 ± 0.44	<1.2	<18

		Cr	Zr	Hf	Ta
*Graphites*					
1	2 (1.00)	<5	<500	<0.18	<0.32
2	63 (0.736)	32.1 ± 3.0	<140	3.9 ± 0.4	<0.6
3	8 (0.715)	22.2 ± 2.2	<90	<0.11	0.221 ± 0.061
4	6 (0.626)	97 ± 9	<500	2.14 ± 0.24	<4
5	16 (0.567)	119 ± 11	<700	6.9 ± 0.7	3.27 ± 0.68
6	100 (0.567)	230 ± 20	<500	5.8 ± 0.6	<2.1
*Semi-graphites*					
7	3 (0.555)	<4	<130	<0.16	<0.8
8	83 (0.543)	5.8 ± 1.6	<250	<0.4	<0.7
*Meta-anthracites*					
9	1 (0.371)	34 ± 5	<220	<0.30	<0.7
10	9 (0.269)	16.0 ± 1.5	<150	0.95 ± 0.09	<0.4

**Table 3:** Results of the analysis of graphite samples

No.	Sample no. (graphitization index)	Content and uncertainty (ppm)			
		La	Ce	Nd	Sm
*Graphites*					
1	2 (1.00)	<1.0	6.9 ± 1.0	<60	<0.10
2	63 (0.736)	<1.4	5.3 ± 1.7	<20	<0.07
3	8 (0.715)	<1.1	8.3 ± 1.2	<18	<0.7
4	6 (0.626)	22.6 ± 2.7	67 ± 9	<50	2.35 ± 0.67
5	16 (0.567)	34.0 ± 2.0	13.1 ± 2.5	<50	5.3 ± 1.1
6	100 (0.567)	39.7 ± 2.2	124 ± 15	<60	4.1 ± 0.9
*Semi-graphites*					
7	3 (0.555)	3.8 ± 0.5	11.3 ± 1.6	<17	<0.07
8	83 (0.543)	2.16 ± 1.1	77 ± 10	<50	2.91 ± 0.46
*Meta-anthracites*					
9	1 (0.371)	5.0 ± 0.5	23.4 ± 3.1	<32	<0.12
10	9 (0.269)	8.9 ± 1.0	29.5 ± 2.6	<31	<1.0

		Eu (ppb)	Tb	Yb	Lu (ppb)
*Graphites*					
1	2 (1.00)	<21	<0.24	<0.8	<60
2	63 (0.736)	62 ± 12	<17	0.67 ± 0.17	<40
3	8 (0.715)	27 ± 6	<0.09	<0.4	<33
4	6 (0.626)	291 ± 88	<0.24	1.60 ± 0.48	191 ± 39
5	16 (0.567)	2050 ± 80	2.2 ± 0.3	5.1 ± 1.0	453 ± 65
6	100 (0.567)	1650 ± 230	2.5 ± 0.5	6.4 ± 0.9	450 ± 65
*Semi-graphites*					
7	3 (0.555)	105 ± 9	<0.15	0.36 ± 0.11	40 ± 13
8	83 (0.543)	920 ± 140	0.91 ± 0.25	3.92 ± 0.60	165 ± 30
*Meta-anthracites*					
9	1 (0.371)	297 ± 97	<0.7	2.02 ± 0.41	180 ± 36
10	9 (0.269)	287 ± 29	<0.5	1.58 ± 0.55	178 ± 29

**Table 4:** Data for the IAEA RM SL1 quantitative evaluation of the analysis accuracy

Element	Certified concentration (ppm)	Deviation of the result of analysis (%)	*Z*-score
Na	1700	−11.7	−1.97
Rb	113	−17.4	−1.23
Cs	7.0	−9.71	−1.02
Sc	17.3	−4.6	−0.70
Th	14	10.7	1.34
Fe (%)	6.740	2.08	0.38
Co	19.8	−5.0	−0.75
Sb	1.31	87	1.58
Cr	104	−2.88	0.38
Ta	1.58	−31	−2.30
La	52.6	−11.2	−1.95
Ce	117	1.71	−0.08
Sm	9.25	−6.05	−0.91
Tb	1.4	−4.29	−0.25
Yb	3.42	−17.0	−0.79
Lu	0.54	35.2	0.80

The elements determined in all analyzed samples are Sc, Fe and Ce. For Zr, Zn, Te and Nd only the lower determination limits could be evaluated. In sample 2 (Sri Lanka) the lowest values of: Sc, Th, Co, Ta, La, Eu, and second lowest: Rb, Fe, Cr, Hf, Ce, Sm, Yb are observed. Also the other graphites (63—Tsavo, Kenya and 8—Passawa, Bawaria) have low level of impurities, comparable with semi-graphite 3 (Keiserberg, Austria) and meta-anthracite 9 (Sonora-Mexico). The semi-graphite 3 have slightly higher amounts of As, Sb, and the meta-anthracite 9 of REE—La, Ce, Eu, Yb, Lu. The two graphites with lowest graphitization index (samples 16—Witostowice, Poland and 100—Młoty, Poland) are most abundant in non-carbon elements. These elements are: alkali metals—Na and Rb (also Cs in sample 100), Sc and actinides—Th, U, refractory metals—Cr, Hf, and REE—La, Sm, Eu, Tb, Yb, Lu (also Ce in sample 100).

Based on the contents of the determined elements in individual samples certain regularity in their presence can be observed. For example, in samples classified as the graphite group, a clear tendency of an increase in content of such elements as Rb, Th, U, Cr, Hf and REE with the descending graphitization index can be observed. In the case of semi-graphite one realizes a significant difference in the contents of Cs, Th, Sc, Cr, Fe, Hg, Sb, and REE between the two samples. The sample of semi-graphite from the contact metamorphism (83) contains more Th, Sc, Cr, Fe, Hg, and REE than sample (3) occurring in phyllitic rocks, that in turn contains more Cs. In the group of meta-anthracites, vice versa, the sample of contact metamorphism (9) has less Sc, Cr, Fe, Co, Ag and Hg, and more Hf, while no essential differences in the REE are observed in these samples.

One may pay attention to the high content of Zn, As, and Sb in the sample no. 2 and Hg in the sample no. 83. They depart from the results of remaining samples and are, perhaps, caused by contamination of samples in their processing, before irradiation.

**Fig. 1:**
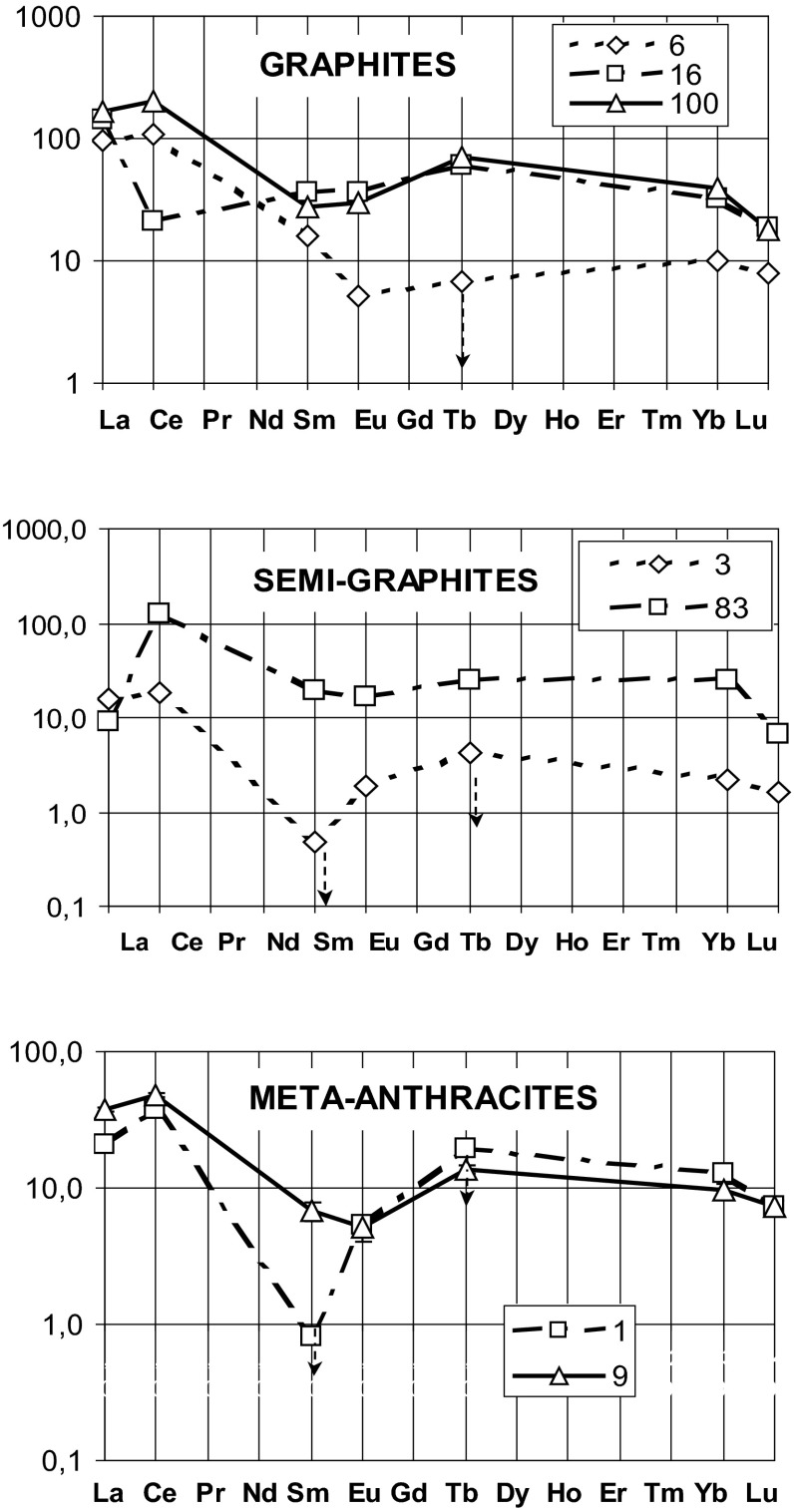
Chondrite normalized patterns of REE for some of the analysed samples. *Points with arrows* show values of the element determination limit for the given sample

Comparison of the chondrite normalized patterns (Fig. 1) for groups of the analyzed samples shows a decrease of REE content with the lowering graphitization index, going from graphites to meta-anthracites. Following the [16] rezoning it could be explained by the property of graphite crystal lattice to efficiently accommodate (intercalate) REE atoms and thus better concentrate these elements in samples of higher graphitization. However, it may also be a result of relatively high level of the impurities in the graphite samples selected for comparison.

There were observed also some correlations between certain pairs of elements for the whole set of samples. However, it is not possible to draw on these basis distinct or clear correlations between amounts of TE and the origin of individual graphites and their age (precambrian, carboniferous), also because of the limited number of samples.

## Conclusions

The presented results are a rare set of TE (among them alkaline metals, actinides and other metals) contents in pure natural graphitic materials from a remarkably different deposits. The contents are confronted with values of the graphitization index determined for these materials. Based on this confrontation certain preliminary observations could be drawn, i.e. the increasing content of some elements in samples of graphite with the descending graphitization index. However, more decisive findings were restricted due to not sufficient number of samples. A more representative research should be continued for more clear observations.
